# Socioeconomic factors associated with chlamydia and gonorrhea positivity in the United States, 2022–2024

**DOI:** 10.1186/s12889-026-27715-4

**Published:** 2026-05-26

**Authors:** Larry He, Chirag G. Patel, Yamir Salabarría-Peña, Guoyu Tao

**Affiliations:** 1 Oak Ridge Institute for Science and Education, Division of STD Prevention, National Center for HIV, Viral Hepatitis, STD, and TB Prevention, Centers for Disease Control and Prevention, Atlanta, GA USA; 2 Division of STD Prevention, National Center for HIV, Viral Hepatitis, STD, and TB Prevention, Centers for Disease Control and Prevention, 1600 Clifton Road NE, Mailstop H24-4, Atlanta, GA 30329-4018 USA

**Keywords:** Chlamydia, Gonorrhea, STI positivity, Housing, Education, Socioeconomic factors

## Abstract

**Background:**

Socioeconomic factors (SEF) can be associated with health outcomes. Understanding the relationships among SEF, demographics, and sexually transmitted infection (STI) positivity can help identify areas to improve STI prevention and care with greatest impact.

**Methods:**

We identified chlamydia and gonorrhea tests from January 2022 to April 2024 for patients aged 15-44 years in the Truveta electronic health record dataset. We estimated the positivity of chlamydia and gonorrhea and stratified by patient demographics (age, sex, race and ethnicity) and SEF measures (recent eviction and educational achievement).

**Results:**

Among 1,799,427 urogenital chlamydia and 1,795,115 urogenital gonorrhea tests conducted on patients aged 15-44 years from January 2022 to April 2024, the overall chlamydia and gonorrhea positivity were 5.43% and 1.86%, respectively. Patients with no college education on record had higher urogenital chlamydia and gonorrhea positivity than those with 4-year college (5.47% vs. 2.90% for chlamydia and 2.05% vs. 0.66% for gonorrhea). Patients with recent eviction had higher urogenital chlamydia and gonorrhea positivity than those without (7.31% vs. 4.83% for chlamydia and 3.51% vs. 1.67% for gonorrhea).

**Conclusion:**

The association between housing insecurity, lower educational attainment, and higher positivity for chlamydia and gonorrhea underscores the need for intersectoral targeted efforts to address challenges contributing to suboptimal STI infections.

## Introduction

Chlamydia and gonorrhea are the two most reported sexually transmitted infections (STIs) in the United States of America (USA). In 2023, over 1.6 million cases of chlamydia and 600,000 cases of gonorrhea were reported to the Centers for Disease Control and Prevention (CDC) [1]. Some subpopulations, such as men who have sex with men (MSM) and people residing in rural areas, are disproportionately affected by chlamydia and gonorrhea. These differences in outcomes are influenced by a range of social, economic, and structural conditions that affect exposure to risk and access to prevention and care [2]. In fact, the relationship between health outcomes (such as STI rates by county) and underlying life conditions is well documented in the literature and continues to be an important focal point in STI prevention [3]. Therefore, it is important to evaluate how and which of these non-medical factors are related to STI infections, as well as to examine how access to and quality of STI care can contribute to more impactful interventions among disproportionately impacted subpopulations for better STI prevention and control.

Previous research has identified associations between STI rates from surveillance reports and a range of county-level social and demographic characteristics. For instance, higher gonorrhea case rates have been observed in counties with greater proportions of residents living below the poverty line [4]. Similarly, county-level eviction rates have been linked with chlamydia and gonorrhea case rates across the USA, even after adjusting for relevant covariates [5]. Additionally, structural barriers such as unstable housing, limited transportation, and lack of access to affordable healthcare—often associated with lower income and limited educational opportunities—have been linked to reduced engagement in protective sexual health behaviors, including condom use [6]. These findings highlight the importance of considering broader socioeconomic factors (SEF) that may be related to STI case rates.

The two studies previously mentioned that examined the relationship between STIs case rates and socioeconomic factors actually relied on ecological data with area-level demographics, rather than patient-level information [4, 5]. Our study aimed to build on this research by leveraging patient-level data linked with individual demographics and socioeconomic variables to better reflect individual experiences and more directly to their own STI infections. Our study estimated chlamydia and gonorrhea test positivity overall, by demographic characteristics, and by patient-level socioeconomic measures and examined correlations between chlamydia and gonorrhea testing positivity and socio-demographic variables. This approach supported a more nuanced understanding of the association between STI infections and socio-demographic variables and may inform public health strategies that aim to address the underlying socioeconomic factors.

## Methods

### Database used in this study

We used Truveta data, a dataset consisting of outpatient and inpatient electronic health records (EHR) in the United States, in this study. The data covers over 120 million patients receiving care at over 20,000 clinics and 900 hospitals in the US. The Truveta data were constructed to have more than 80 files (tables) and all data are expertly de-identified, accurately linked, and cleaned to enable longitudinal research. For example, the Person table has person ID, patient’s age, sex, and race and ethnicity; the Labresult table has person ID, encounter ID, date of lab test, and many lab related information; the Condition table and Procedure table have patient’s medical conditions and medical procedures during their healthcare encounters, respectively; and the SDOH table has several SEF measures, which were originally from the LexisNexis data [7]. Some files, such as the Location table that has patient’s region and state location, were recently added.

The Truveta data at CDC are updated several times per year. Because the same person will be assigned different person ID after the update, researchers need to save all required information (subsets) into their own folders for further data analyses before the update. If the researchers did not save required information, there was a limited way to get any information related to person ID from the updated Truveta data. For this study, we only saved the information from the following tables: Person, Labresult, and SDOH.

To assess the representative data that can be generalized to the US population, Truveta research team had evaluated the data and stated that at the state level, Truveta adheres to the benchmark set by the Center for Medicare & Medicaid Services (CMS) under their qualified entity framework, where representativeness is based on whether Truveta Data includes at least 10% of the state’s population of covered lives. Based on evaluation results, Truveta meets this benchmark in 26 states [8]. 

### SEF measures

Several de-identified patient-level SEF measures were added in the Truveta data through a partnership with LexisNexis Health Solutions [9]. Drawn from government, business, and other records, these SEF measures were constructed once for each patient from January 2022 to April 2024. The SEF measures utilized in this study are educational achievement and recent eviction. Although annual income and other SEF data were available, we excluded them due to high proportions of missing data. For example, annual income was missing for about 67.3% of patients. The Truveta data we received included four main categories of educational attainment: (1) ‘no college on record’, (2) ‘some colleges did not finish’, (3) ‘2-year college degree’, and (4) ‘4-year college degree’. Noted that a person in the process of obtaining a degree was categorized in either 2-year college degree or 4-year college degree. A person was defined as having a recent eviction if the length of time since their most recent recorded eviction was less than 60 months and the length of time in their recorded current residence was less than 60 months.

The patient-level SEF measures in Truveta data have not been updated after April 2024, and the SEF measures from LexisNexis were no longer linked to the Truveta data after 2024. Several new area-level SEF measures from other resources will be linked to the Truveta soon.

### Chlamydia and gonorrhea positivity

In the Truveta laboratory result file, chlamydia or gonorrhea tests performed during January 2022-April 2024 among patients aged 15–44 years were initially identified with Logical Observation Identifiers Names and Codes (LOINC) codes. One of the main reasons we limited the timespan from January 2022 to April 2024 was that the SEF variables collected from LexisNexis might be the best to represent patient’s social economic condition during that period. Most chlamydia or gonorrhea tests (98.2%) were nucleic acid amplification tests during the study period in the Truveta data. According to LOINC codes and the laboratory result description for each test, chlamydia and gonorrhea tests were excluded from further analysis if they were coded with blood, serum, or conjunctive specimen or with antigen tests. Of the remaining tests, tests were further excluded if their results were invalid. Only positive or negative results of chlamydia or gonorrhea tests were considered valid results; all other test results, such as undetermined or tests not performed, were considered invalid.

Of those chlamydia and gonorrhea tests with valid results, we further grouped them by testing date and by specimen type for each patient. Rectal or anal specimens were classified as “rectal”, pharyngeal, oral, or throat specimens as “pharyngeal”, and vaginal, endocervical, urine, urethral specimens, or non-specific specimen as “urogenital”. For a given testing date for each patient, there might be duplicated tests for each specimen type. For example, for a given date for a given patient, there might be two chlamydia urogenital tests: one with positive result and one with negative result, two chlamydia rectal tests: both with positive results, and three chlamydia pharyngeal tests: all of three with negative results. In such cases, only one chlamydia rectal test was included, one chlamydia pharyngeal test was included, and no chlamydia urogenital test was included because the results of duplicated tests for chlamydia urogenital were inconsistent.

Repeating testing or test-of-care may affect the positivity estimate. To address this concern, we further grouped all testing dates into episodes for each patient. The episodes were created with the following two conditions. First, there might be more than one testing date in each episode and among all testing dates in the given episode at least two testing dates were within 28 days if there were more than one testing date. Second, the last testing date in the previous episode was at least 28 days prior to the first testing date in the current episode. For each episode, only chlamydia and gonorrhea tests at the first testing date were included in the final data analysis.

Chlamydia and gonorrhea positivity for a given specimen was defined as the total number of tests that were positive at the given specimen divided by the total number of tests that were either positive or negative for the given specimen.

### Data analyses

Patient’s chlamydia and gonorrhea tests were linked with their demographic information and SEF measures. Ethnicity data included two categories: “Hispanic or Latino” and “Non-Hispanic or Latino”. Merging race and ethnicity in this study involved a stepwise process whereby patients with Hispanic ethnicity were first classified as Hispanic or Latino, regardless of the presence or absence of race data. Patients noted to be non-Hispanic were categorized based on race. For demographic analyses, chlamydia and gonorrhea positivity were stratified by age at time of test (15–19 years, 20–24 years, 25–29 years, 30–34 years, 35–39 years, and 40–44 years), sex (male, female), and race/ethnicity (non-Hispanic American Indian or Alaska Native, non-Hispanic Asian, non-Hispanic Black or African American, Hispanic or Latino, non-Hispanic Native Hawaiian or Other Pacific Islander, non-Hispanic White, and non-Hispanic other race). The final categories for race/ethnicity were mutually exclusive. Likewise, for SEF analyses, chlamydia and gonorrhea positivity were stratified by each SEF measure.

All positivity rates were calculated with 95% confidence intervals (95% CI). The χ^2^ test was used to compare chlamydia and gonorrhea positivity by each demographic characteristic and by each socioeconomic factor. For chlamydia and gonorrhea tests with missing value for a given demographic variable or socioeconomic factor, a missing category was included in the χ^2^ test. Statistical significance was considered if there was lack of overlap of confidence intervals of chlamydia or gonorrhea positivity, when two categories were compared for the given demographic variable or socioeconomic factor.

Some patients may have more than one chlamydia or gonorrhea test, and those test results may be correlated during the study period. In addition, some demographic variables and SEF variables might be highly correlated too, such as patient’s age and education level, and those correlations may affect how to interpret the association between chlamydia and gonorrhea positivity and the given demographic or SEF variable. To address those concerns, the generalized estimating equations (GEE) models were used to identify the association between chlamydia or gonorrhea positivity and SEFs and demographic variables.

Structured Query Language (SQL) was used for data preparation and SAS version 9.4 (Cary, NC) was used for statistical analyses in this study.

### Human subjects

This study performs analysis of de-identified electronic health records (EHR) data accessed via Truveta Studio. De-identification is attested to through expert determination in accordance with the HIPAA Privacy Rule. This study was reviewed by the CDC IRB, deemed research not involving human subjects, and was conducted consistently with applicable federal law and CDC policy.

## Results

Among 2,858,682 chlamydia tests and 2,796,693 gonorrhea tests with valid specimen, 2,818,063 (98.6%) and 2,784,900 (99.6%) had valid testing results, respectively. Of those chlamydia and gonorrhea tests with valid specimen and valid testing results, 1,894,180 unique chlamydia tests and 1,982,181 gonorrhea tests were created based on patient ID, testing date, specimen, and testing results. Of those tests, 1332 chlamydia tests and 915 gonorrhea tests were excluded due to the inconsistent testing results. Of the remaining 1,892,848 chlamydia tests among 1,319,898 patients with 1,877,140 testing dates, 1,816,191 chlamydia tests with 1,802,571 episodes were included for further analysis. Similarly, of the remaining 1,981,266 gonorrhea tests among 1,339,936 patients with 1,908,077 testing dates, 1,898,467 gonorrhea tests with 1,829,994 episodes were included for further analysis.

Of 1,816,191 chlamydia tests and 1,898,467 gonorrhea tests, 99.1% and 94.6% were with urogenital specimen, 0.8% and 0.9% with pharyngeal specimen, and 0.1% and 4.5% with rectal specimen. Chlamydia and gonorrhea positivity by specimen overall and stratified by sex were presented in Table 1.

**Table 1:** Chlamydia and gonorrhea positivity by specimen type and by sex among patients aged 15–44 years during January 2022- April 2024, Truveta

Specimen type	Chlamydia		Gonorrhea	
	Number of Tests	Positivity (%) (95% CI)	Number of Tests	Positivity (%) (95% CI)
Urogenital				
Overall	1,799,427	5.43 (5.39–5.46)	1,795,115	1.86(1.84–1.88)
Male	340,658	8.72 (8.63–8.82)	330,298	5.24 (5.17–5.32)
Female	1,457,133	4.66 (4.62–4.69)	1,463,196	1.09 (1.08–1.11)
Pharyngeal				
Overall	14,047	1.35 (1.16–1.54)	17,747	2.59 (2.36–2.83)
Male	9,938	1.06 (0.86–1.26)	10,370	3.38 (3.03–3.72)
Female	4,061	2.09 (1.65–2.53)	7,322	1.50 (1.22–1.78)
Rectal				
Overall	2,717	5.12 (4.29–5.94)	85,605	1.86 (1.77–1.95)
Male	2,310	5.11 (4.21–6.01)	15,364	5.02 (4.67–5.36)
Female	382	5.50 (3.21–7.78)	70,176	1.18 (1.10–1.26)

Among 1,799,427 urogenital chlamydia and 1,795,115 gonorrhea tests conducted on 1,319,898 and 1,339,936 patients aged 15–44 years from January 2022 to April 2024, the overall chlamydia and gonorrhea positivity rates for urogenital specimen were 5.43% (95% CI = 5.39%, 5.46%) and 1.86% (95% CI = 1.84%, 1.88%), respectively (Table 2). Men accounted for 18.93% of urogenital chlamydia tests and 18.40% of urogenital gonorrhea tests and had significantly higher positivity rates compared to women. Chlamydia and gonorrhea positivity rates were significantly higher among younger patients than those aged 40–44 years. Urogenital chlamydia positivity was the highest among patients who identified as Non-Hispanic Native Hawaiian or Other Pacific Islander at 8.70% (95% CI = 8.20%, 9.20%), followed by Non-Hispanic Black or African American at 8.28% (95% CI = 8.20%, 8.35%), Non-Hispanic American Indian or Alaska Native at 6.91% (95% CI = 6.44%, 7.39%), Hispanic or Latino at 5.61% (95% CI = 5.53%, 5.68%), Non-Hispanic White at 3.82% (95% CI = 3.78%, 3.87%), and Non-Hispanic Asian at 2.54% (95% CI = 2.42%, 2.65%). For urogenital gonorrhea, positivity was the highest among patients identifying as Non-Hispanic Black or African American at 3.85% (95% CI = 3.79%, 3.90%), followed by Non-Hispanic American Indian or Alaska Native at 2.10% (95% CI = 1.82%, 2.37%), Non-Hispanic Native Hawaiian or Other Pacific Islander at 1.75% (95% CI = 1.51%, 1.98%), Hispanic or Latino at 1.30% (95% CI = 1.26%, 1.34%), Non-Hispanic White at 1.06% (95% CI = 1.04%, 1.09%), and Non-Hispanic Asian at 0.47% (95% CI = 0.42%, 0.52%).

**Table 2:** Chlamydia and gonorrhea urogenital positivity by demographic and socioeconomic factors among patients aged 15–44 years During January 2022- April 2024, Truveta

Demographic/Socioeconomic Factor	Chlamydia		Gonorrhea	
	Number of Tests (%)	Positivity (%) (95% CI)	Number of Tests (%)	Positivity (%) (95% CI)
Overall	1,799,427	5.43 (5.39–5.46)	1,795,115	1.86 (1.84–1.88)
Calendar year tests performed				
2022	766,038	5.63 (5.58–5.68)	735,268 (40.96)	2.15 (2.12–2.19)
2023	783,234	5.38 (5.33–5.43)	802,438 (44.70)	1.74 (1.71–1.77)
2024	250,155	4.94 (4.86–5.03)	257,409 (14,34)	1.38 (1.33–1.42)
Sex				
Male	340,658 (18.93)	8.72 (8.63–8.82)	330,298 (18.40)	5.24 (5.17–5.32)
Female	1,457,133 (80.98)	4.66 (4.62–4.69)	1,463,196(81.51)	1.09 (1.08–1.11)
Missing	1,636(0.09)	3.73 (2.81–4.65)	1,621(0.09)	2.47 (1.71–3.22)
Age in Years				
15–19	215,204 (11.96)	12.23 (12.09–12.37)	207,652 (11.57)	3.47 (3.39–3.54)
20–24	444,599 (24.71)	8.20 (8.12–8.28)	441,134 (24.57)	2.27 (2.22–2.31)
25–29	405,094 (22.51)	4.41 (4.34–4.47)	408,427 (22.75)	1.57 (1.53–1.61)
30–34	355,449 (19.75)	2.76 (2.71–2.82)	359,098 (20.00)	1.36 (1.32–1.40)
35–39	234,864 (13.05)	2.04 (1.98–2.09)	235,890 (13.14)	1.28 (1.24–1.33)
40–44	144,217 (8.01)	1.64 (1.58–1.71)	142,914 (7.96)	1.27 (1.21–1.32)
Race/Ethnicity				
Non-Hispanic American Indian or Alaska Native	10,818 (0.60)	6.91 (6.44–7.39)	10,391 (0.58)	2.10 (1.82–2.37)
Non-Hispanic Asian	75,606 (4.20)	2.54 (2.42–2.65)	76,830 (4.28)	0.47 (0.42–0.52)
Non-Hispanic Black or African American	474,268 (26.36)	8.28 (8.20–8.35)	466,624 (25.99)	3.85 (3.79–3.90)
Hispanic or Latino	339,948 (18.89)	5.61 (5.53–5.68)	342,276 (19.07)	1.30 (1.26–1.34)
Non-Hispanic Native Hawaiian or Other Pacific Islander	12,249 (0.68)	8.70 (8.20–9.20)	12,193 (0.68)	1.75 (1.51–1.98)
Non-Hispanic Other Race	48,636 (2.70)	5.15 (4.95–5.34)	50,240 (2.80)	1.64 (1.53–1.75)
Non-Hispanic White	738,464 (41.04)	3.82 (3.78–3.87)	735,968 (41.00)	1.-6 (1.04–1.09)
missing	99,438(5.53)	4.86(4.73–4.99)	100,593 (5.60)	1.49 (1.41–1.56)
Highest Education Level				
No college on record	1,033,522 (57.44)	5.47 (5.43–5.52)	1,017,929 (56.71)	2.05 (2.02–2.07)
Some college but did not complete	112,436 (6.25)	3.69 (3.58–3.80)	109,571 (6.10)	1.36 (1.29–1.42)
2-year college degree	107,097 (5.95)	4.97 (4.84–5.10)	109,587 (6.10)	1.33 (1.27–1.40)
4-year college degree	217,648 (12.10)	2.90 (2.83–2.97)	218,053 (12.15)	0.66 (0.63–0.70)
missing	328,724 (18.27)	7.68 (7.59–7.77)	339,975 (18.94)	2.39 (2.34–2.44)
Recent Eviction				
Yes	50,315 (2.80)	7.31 (7.08–7.54)	47,671 (2.66)	3.51 (3.34–3.67)
No	1463,523 (81.33)	4.83 (4.79–4.8)	1,453,439 (80.97)	1.67 (1.65–1.69)
missing	285,589 (15.87)	8.16 (8.06–8.26)	294,005 (16.38)	2.51 (2.46–2.57)

Urogenital chlamydia positivity was the highest among patients with no college education on record (5.47%, 95% CI = 5.43%, 5.52%) and the lowest among those with a 4-year college degree (2.90%, 95% CI = 2.83%, 2.97%). For urogenital gonorrhea, the highest positivity was among patients with no college education on record at 2.05% (95% CI = 2.02%, 2.07%), while the lowest rate was observed among those with a 4-year college degree at 0.66% (95% CI = 0.63%, 0.70%). Patients with recent eviction had notably higher positivity: 7.31% (95% CI = 7.08%, 7.54) for urogenital chlamydia and 3.51% (95% CI = 3.34%, 3.67%) for urogenital gonorrhea, compared to those without a recent eviction 4.83% (95% CI = 4.79%, 4.86%) for chlamydia and 1.67% (95% CI = 1.65%, 1.69%) for gonorrhea (Table 2).

**Fig. 1:**
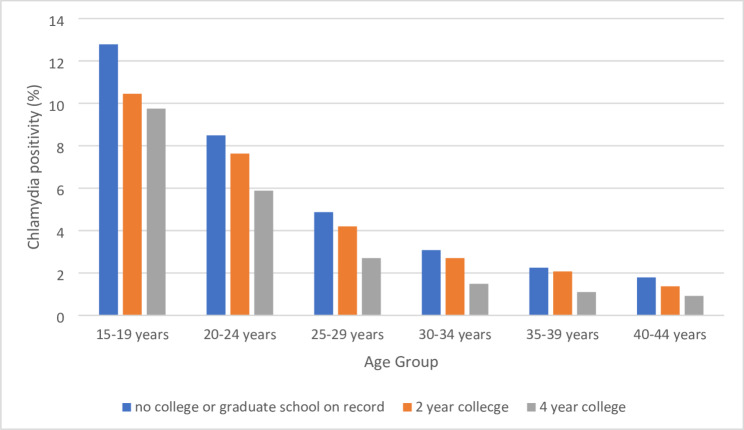
Chlamydia positivity by education level stratified by age group among patients aged 15–44 years from January 2022-April 2024, Truveta

**Fig. 2:**
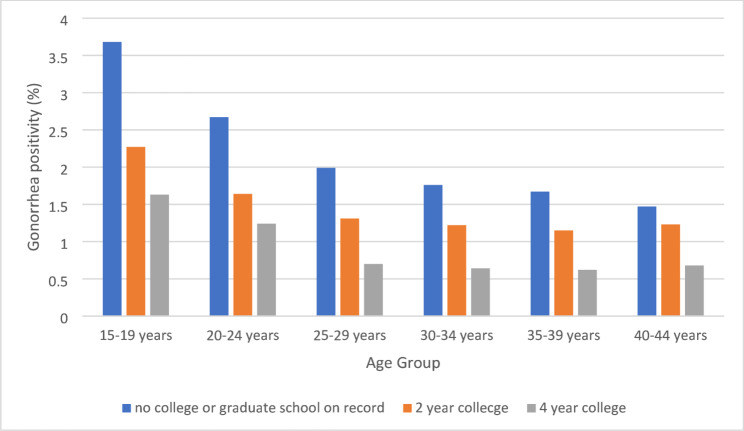
Gonorrhea positivity by education level stratified by age group among patients aged 15–44 years from January 2022- April 2024, Truveta

When stratified by age, patients with no college education on record had the highest urogenital chlamydia and gonorrhea positivity across most age groups (Figs. 1 and 2). Across all age groups, patients with a 4-year college degree consistently had the lowest urogenital chlamydia and gonorrhea positivity.

The GEE model showed that, after adjusted for demographic variables, patients with recent eviction had higher urogenital chlamydia and gonorrhea positivity than those without recent eviction and patients with less education had higher urogenital chlamydia and gonorrhea positivity than those with more education (Table 3).

**Table 3:** Association between chlamydia or gonorrhea urogenital positivity and demographic and socioeconomic factors among patients aged 15–44 years during January 2022- April 2024, from the GEE models, Truveta

Demographic/Socioeconomic Factor	Chlamydia		Gonorrhea	
	Number of Tests (%)	Adjusted Odds Ratio (95% CI)	Number of Tests (Proportion)	Adjusted Odds Ratio (95% CI)
Sex				
Male	340,658 (18.93)	referral	330,298 (18.40)	referral
Female	1,457,133 (80.98)	0.50 (0.49–0.50)	1,463,196 (81.51)	0.21 (0.20–0.21)
missing	1,636 (0.09)	0.66 (0.49–0.87)	1,621 (0.09)	0.77 (0.55–1.07)
Age in Years				
15–19	215,204 (11.96)	8.63 (8.25–9.02)	207,652 (11.57)	2.97 (2.80–3.15)
20–24	444,599 (24.71)	5.80 (5.56–6.06)	441,134 (24.57)	2.15 (2.03–2.27)
25–29	405,094 (22.51)	2.99 (2.86–3.13)	408,427 (22.75)	1.49 (1.41–1.58)
30–34	355,449 (19.75)	1.85 (1.77–1.94)	359,098 (20.00)	1.28 (1.21–1.36)
35–39	234,864 (13.05)	1.33 (1.26–1.40)	235,890 (13.14)	1.15 (1.08–1.22)
40–44	144,217 (8.01)	referral	142,914 (7.96)	referral
Race/Ethnicity				
Non-Hispanic American Indian or Alaska Native	10,818 (0.60)	1.88 (1.73–2.04)	10,391 (0.58)	1.90 (1.63–2.20)
Non-Hispanic Asian	75,606 (4.20)	0.76 (0.73–0.80)	76,830 (4.28)	0.47 (0.42–0.52)
Non-Hispanic Black or African American	474,268 (26.36)	2.17 (2.13–2.21)	466,624 (25.99)	3.22 (3.13–3.32)
Hispanic or Latino	339,948 (18.89)	1.40 (1.37–1.43)	342,276 (19.07)	1.11 (1.06–1.15)
Non-Hispanic Native Hawaiian or Other Pacific Islander	12,249 (0.68)	2.41 (2.24–2.59)	12,193 (0.68)	1.69 (1.46–1.96)
Non-Hispanic Other Race	48,636 (2.70)	1.31 (1.25–1.37)	50,240 (2.80)	1.38 (1.28–1.49)
Non-Hispanic White	738,464 (41.04)	referral	735,968 (41.00)	referral
missing	99,438 (5.53)	1.17 (1.13–1.21)	100,593 (5.60)	1.14 (1.07–1.21)
Highest Education Level				
No college on record	1,033,522 (57.44)	referral	1,017,929 (56.71)	referral
Some college but did not complete	112,436 (6.25)	0.81 (0.78–0.84)	109,571 (6.10)	0.77 (0.72–0.81)
2-year college degree	107,097 (5.95)	0.92 (0.89–0.95)	109,587 (6.10)	0.72 (0.68–0.76)
4-year college degree	217,648 (12.10)	0.65 (0.64–0.67)	218,053 (12.15)	0.40 (0.38–0.42)
missing	328,724 (18.27)	0.83 (0.79–0.87)	339,975 (18.94)	0.78 (0.72–0.85)
Recent Eviction				
Yes	50,315 (2.80)	referral	47,671 (2.66)	referral
No	1463,523 (81.33)	0.75 (0.72–0.77)	1,453,439 (80.97)	0.59 (0.56–0.62)
missing	285,589 (15.87)	0.90 (0.85–0.96)	294,005 (16.38)	0.76 (0.69–0.84)

## Discussion

The study is among the first to examine the association between patient-level educational achievement and eviction with chlamydia and gonorrhea positivity using a large, multi-year EHR dataset. Our findings reveal that both lower educational attainment and indicators of housing instability were associated with higher chlamydia and gonorrhea positivity at the patient-level, underscoring the role of personal-level socioeconomic factors in shaping STI outcomes.

Educational attainment, a fundamental factor in socioeconomic status, was strongly associated with STI positivity in our analysis, indicating similar pattern in previous studies [10–12]. When stratified by age, patients without a 4-year college degree – particularly those with no college experience – had significantly higher positivity. These findings suggest that barriers to college educational attainment, which often reflect broader systemic barriers such as economic hardship or structural factors, may have implications for STI health outcomes. This aligns with existing evidence linking lower educational attainment with reduced access to preventive health resources and structural barriers to consistent engagement in behaviors such as consistent condom use and routine STI screening [13–15]. 

Additionally, differences in STI positivity in this study’s cohort by recent eviction status may reflect underlying differences in housing stability and socioeconomic security. These patterns may reflect that patients experiencing housing instability had reduced access to consistent healthcare, limited privacy for sensitive health needs, and many barriers to preventive health engagement [5, 16–19]. 

Our findings of positivity by demographic group were generally consistent with prior studies. The chlamydia and gonorrhea positivity rates among young women and the peak age for positivity that we observed were similar to previous studies [20, 21]. We observed the highest positivity for both chlamydia and gonorrhea among younger patients, particularly those aged 15–19 years, among non-Hispanic Black or African American patients who had the highest positivity for gonorrhea, among American Indian or Alaska Natives, and among Native Hawaiian or Other Pacific Islanders who had the highest positivity for chlamydia. Our results reconfirmed that disparities exist among people with different SEF status and that SEFs are related to STD rates [3]. AS discussed in the previous study, lower SEF status is also related to community segregation, lack of access to healthcare, and higher incarceration rates. Our results suggest that healthcare providers, STI clinics, and local health departments that serve many of those minority populations might tailor public health interventions not only to improve patient’s access to healthcare and to provide timely and quality care for STI but also engage community-level activities that are explicitly tied to reducing disparities. For example, healthcare providers need to be aware of SEF-related STI rates and may provide enhanced services when they have patient’s SEF information in their clinics. Outside clinical environments, they may participate in clinical training of avoiding STI stigmatization, school-based screening/education programs, or community events that try to improve community health in general, such as HIV, reproductive health, and stress reduction.

This study has several limitations. First, Truveta data, while extensive, may be not nationally representative because Truveta data is derived from 30 health systems across the United States. Second, Truveta’s data processing pipeline that convert original medical information to standardize data may introduce potential misclassification of data attributes [7]. For example, the proportion of chlamydia and gonorrhea tests that were pharyngeal or rectal in this study was much lower than the previous study [24]. The difference might be due to misclassification or difference in patient populations served in two datasets. Fortunately, urogenital tests were the most tests in both studies, our results related to urogenital chlamydia and gonorrhea positivity were considered as reliable. Third, because both SEF measures (education level and recent eviction) had high proportion with missing, the interpretation of the results needs caution. Furthermore, the SEF may be insufficiently defined due to the group definitions having been made by LexisNexis. For example, the 60 months range for recent eviction is longer than the timespans used for recent eviction from past research of 2 or fewer years; the rationale for the extended time span may have been influenced by de-identification requirements [22]. Fourth, multiple SEFs, such as household income and individual income, were excluded from the analysis due to missing data that are known to be correlated with STI positivity, housing instability, and educational attainment [3]. Fifth, education data lacked detail on enrollment status or completion timing. Patients who recently completed degree programs were grouped with those who earned degrees long ago, potentially conflicting with the short- and long-term effects of educational attainment on health outcomes. Graduate education was not distinguished from undergraduate education, weakening interpretability due to graduate education being linked to higher lifetime income [23]. Sixth, the mismatch between SEF assignment and test dates may introduce further uncertainty, especially when there is a significant lag between testing and SEF data capture. Finally, this study also used race and ethnicity to measure patients’ demographics. However, the accuracy of these variables depended on how and by whom the information was entered. For example, data entered directly by patients through an online portal is more likely to be accurate, whereas entries made during emergency department registration may rely on staff observation and, in some cases, assumptions [24]. 

## Conclusion

Our findings had public health implications. Our study suggests that housing insecurity and educational attainment may be associated with chlamydia and gonorrhea test positivity at the patient-level. These results underscore the critical role of socioeconomic conditions in shaping STI outcomes. Strengthening sexual health requires a comprehensive public health approach that considers broader environments in which people live, learn, and seek care [25]. This includes tailored outreach and coordinated efforts across sectors such as public health, health care, education, and housing to support more effective prevention, screening, and treatment of chlamydia and gonorrhea. Until SEF disparities are addressed, the reduction of STI disparities cannot be guaranteed. 

## Data Availability

The Truveta data are owned by Truveta company in USA, CDC has a license to access Truveta data between CDC and Truveta, and the CDC users can have access to Truveta data under the data use agreement. The subset of Truveta data that supports this study is available from the corresponding author upon reasonable request and with permission of Truveta.

## Abbreviations

STIs: Sexually transmitted infections
USA: United States of America
SEF: Socioeconomic factors
CDC: Centers for Disease Control and Prevention
LOINC: Logical Observation Identifiers Names and Codes
CI: Confidence intervals
EHR: Electronic health records

## References

[1] Centersfor Disease Control and Prevention. Sexually transmitted infections surveillance. 2023. In: United States Department of Health and Human Services. ed2025. https://www.cdc.gov/sti/php/from-the-director/announcing-sti-surveillance-2023.html.

[2] Marotta P. Assessing spatial relationships between race, inequality, crime, and gonorrhea and chlamydia in the United States. J Urb Health. 2017;94(5):683–98. 10.1007/s11524-017-0179-5 PMC5610128 28831708

[3] Hogben M, Leichliter JS. Social determinants and sexually transmitted disease disparities. Sex Transm Dis. 2008;35(12):S13–8. 18936725 10.1097/OLQ.0b013e31818d3cad

[4] Moonesinghe R, Fleming E, Truman BI, Dean HD. Linear and non-linear associations of gonorrhea diagnosis rates with social determinants of health. Int J Environ Res Public Health. 2012;9(9):3149–65. 23202676 10.3390/ijerph9093149 PMC3499859

[5] Niccolai LM, Blankenship KM, Keene DE. Eviction from renter-occupied households and rates of sexually transmitted infections: a county-level ecological analysis. Sex Transm Dis. 2019;46(1):63–8. 30148755 10.1097/OLQ.0000000000000904 PMC6289707

[6] Cohn T, Harrison CV. A systematic review exploring racial disparities, social determinants of health, and sexually transmitted infections in Black women. Nurs Women’s Health. 2022;26(2):128–42. 35182482 10.1016/j.nwh.2022.01.006

[7] Truveta. Truveta Data. 2025. https://www.truveta.com/truveta-data/. Access date:12/12/2025.

[8] Truveta. Our approach to data quality. https://resources.truveta.com/hubfs/Whitepapers/Truvetas%20Approach%20to%20Data%20Quality.pdf. Access date: 04/23/2026.

[9] New SDOH data in Truveta Studio [press release]. 2023. https://www.truveta.com/blog/news/new-sdoh-data-2023/. Access date: 12/12/2025.

[10] Workowski KA, Bachmann LH. Centers for disease control and prevention’s sexually transmitted diseases infection guidelines. Clin Infect Dis. 2022;74(Supplement2):S89–94. 35416966 10.1093/cid/ciab1055

[11] Link BG, Phelan J. Social conditions as fundamental causes of disease. J Health Soc Behav. 1995;Spec No:80–94. 7560851

[12] CutlerDM, Lleras-Muney A. Education and health: evaluating theories and evidence. In: National Bureau of Economic Research Cambridge, Mass., USA. 2006. https://www.nber.org/system/files/working_papers/w12352/w12352.pdf.

[13] Cutler DM, Lleras-Muney A. Understanding differences in health behaviors by education. J Health Econ. 2010;29(1):1–28. 19963292 10.1016/j.jhealeco.2009.10.003 PMC2824018

[14] Santelli JS, Lowry R, Brener ND, Robin L. The association of sexual behaviors with socioeconomic status, family structure, and race/ethnicity among US adolescents. Am J Public Health. 2000;90(10):1582. 11029992 10.2105/ajph.90.10.1582 PMC1446372

[15] Zajacova A, Lawrence EM. The relationship between education and health: reducing disparities through a contextual approach. Annu Rev Public Health. 2018;39(1):273–89. 29328865 10.1146/annurev-publhealth-031816-044628 PMC5880718

[16] Widman L, Noar SM, Golin CE, Willoughby JF, Crosby R. Incarceration and unstable housing interact to predict sexual risk behaviours among African American STD clinic patients. Int J STD AIDS. 2014;25(5):348–54. 24060677 10.1177/0956462413505999 PMC4037926

[17] Jennings JM, Leifheit KM. Eviction as a social determinant of sexual health outcomes. In. LWW. 2019;46:69–71. 10.1097/OLQ.0000000000000936 PMC6596301 30365463

[18] Swope CB, Hernández D. Housing as a determinant of health equity: A conceptual model. Soc Sci Med. 2019;243:112571. 31675514 10.1016/j.socscimed.2019.112571 PMC7146083

[19] Aidala AA, Wilson MG, Shubert V, et al. Housing status, medical care, and health outcomes among people living with HIV/AIDS: a systematic review. Am J Public Health. 2016;106(1):e1–23. 26562123 10.2105/AJPH.2015.302905 PMC4695926

[20] Habel MA, Leichliter JS, Torrone E. Exploring chlamydia positivity among females on college campuses, 2008–2010. J Am Coll Health. 2016;64(6):496–501. 26731425 10.1080/07448481.2015.1117470 PMC6738559

[21] Kaufman HW, Gift TL, Kreisel K, Niles JK, Alagia DP. Chlamydia and gonorrhea: Shifting age-based positivity among young females, 2010–2017. Am J Prev Med. 2020;59(5):697–703. 32828583 10.1016/j.amepre.2020.05.023 PMC7903324

[22] Groves AK, Niccolai LM, Keene DE, Rosenberg A, Schlesinger P, Blankenship KM. Housing instability and HIV risk: expanding our understanding of the impact of eviction and other landlord-related forced moves. AIDS Behav. 2021;25(6):1913–22. 33389317 10.1007/s10461-020-03121-8 PMC7778418

[23] Cheah B, Carnevale AP, Wenzinger E. The college payoff more education doesn’t always mean more earnings. 2021. https://cew.georgetown.edu/cew-reports/collegepayoff2021/.

[24] Tao G, Gift TL. High volume and high positivity of chlamydia and gonorrhea tests by anatomic site from a large national laboratory in the United States, 2019 to 2023.Sex Transm Dis. 2025;52(9):523–8. 10.1097/OLQ.0000000000002165 PMC12339204 40178090

[25] United States Department of Health and Human Services. Sexually transmitted infections national strategic plan for the United States. 2021–2025. https://www.hhs.gov/sites/default/files/STI-National-Strategic-Plan-2021-2025.pdf. Access Date:12/12/2025.

